# Optimal cutoff values of intraoperative parathyroid hormone for predicting early and permanent hypoparathyroidism after total thyroidectomy

**DOI:** 10.1007/s00423-025-03619-6

**Published:** 2025-01-31

**Authors:** Pablo Moreno Llorente, Arantxa García Barrasa, Mireia Pascua Solé, José Luis Muñoz de Nova, Marta Alberich Prats

**Affiliations:** 1 Unidad de Cirugía Endocrina, Hospital Universitari de Bellvitge, C/ Feixa Llarga s/n, L’Hospitalet de Llobregat, Barcelona, E-0897 Spain; 2 Departament de Ciències Clíniques, Facultat de Medicina i Ciències de la Salut, Universitat de Barcelona, Barcelona, Spain; 3 Servicio de Cirugía General y Digestivo, Hospital Universitario de La Princesa, Instituto de Investigación Sanitaria Princesa (IIS-IP), Universidad Autónoma de Madrid (UAM), Madrid, Spain

**Keywords:** Hypoparathyroidism, Hypocalcemia, Total thyroidectomy, Intraoperative parathyroid hormone, Diagnostic efficacy

## Abstract

**Purpose:**

Measurement of intraoperative intact parathyroid hormone (ioPTH) levels is a reliable predictor of postsurgical hypocalcemia. We assessed the optimal cutoff values of ioPTH decline for predicting postoperative early and permanent hypoparathyroidism.

**Methods:**

This was a retrospective study of a prospectively maintained database of patients undergoing thyroid surgery in a tertiary care hospital in L’Hospitalet de Llobregat (Barcelona, Spain). All consecutive patients undergoing total thyroidectomy with or without central neck dissection between January 2005 and May 2021 were analyzed. The best cutoff value of the decrease of ioPTH level between PTH assessed after induction of anesthesia and at 10 min after completion of surgery for predicting hypocalcemia was evaluated.

**Results:**

We included 742 patients (mean age 52 years) (thyroid cancer 48%, neck dissection 42%) undergoing total thyroidectomy. Postoperative hypocalcemia was diagnosed in 383 (51.6%) patients, which was transient in 296 (39.9%) and permanent in 87 (11.7%). The optimal cutoff value for predicting transient hypocalcemia was a decline of ioPTH level of ≤ 62.5% (overall efficacy 87%), but calcium supplementation may be indicated in high-risk cutoff values of ≥ 79.9%. In patients with an ioPTH decline ≤ 39%, the probability of postoperative hypocalcemia is extremely unlikely. Patients with declines > 93.7% should be followed very closely since they are high-risk for developing permanent hypoparathyroidism.

**Conclusion:**

The decline of ioPTH, measured as the difference between ioPTH before thyroidectomy and after completion of the surgical procedure is a reliable indicator of the likelihood of postoperative transient hypocalcemia, with optimal cutoff value of 62.5%.

## Introduction

Postoperative hypocalcemia is a common transient event after extensive thyroid surgery due to intraoperative injury of the parathyroid glands or inadvertent surgical ablation of one or more parathyroids. Early hypocalcemia occurs in a variable percentage of patients after total or near total thyroidectomy from less than 5% up to 49%, increasing the length of hospital stay and the morbidity of thyroid surgery [1, 2]. Postoperative hypocalcemia also limits moving towards treating thyroid resections as outpatient procedures. Transient hypoparathyroidism may become permanent in about 10% of patients who will require life-long supplementation therapy and regular follow-up visits, with increased risk of deterioration of health-related quality of life and development of complications and comorbidities [3–5].

Early detection of postsurgical hypoparathyroidism is essential to prevent hypocalcemia-related complications and would allow patients to be discharged from the hospital earlier. Routine postoperative supplementation with calcium/vitamin D has been advocated to facilitate early discharge due to difficulties for identifying patients at risk of clinically significant hypocalcemia [6, 7]. Intraoperative assays of intact parathyroid hormone (ioPTH) were originally introduced in parathyroid surgery showing that proper surgical management of parathyroid diseases was associated with a significant decrease in PTH blood levels [8, 9]. Quick ioPTH measurement has been evaluated in cases of total thyroidectomy as accurate predictor of symptomatic hypocalcemia, with the benefits seen in early management of hypocalcemia, early hospital discharge, rare readmissions, cost savings, and reduced hospital stay [10–14]. In a series of 50 patients with thyroid cancer undergoing total thyroidectomy, a decrease in ioPTH level of at least 62.5% was associated with a sensitivity of 82%, a specificity of 85%, a positive predictive value (PPV) of 60% and a negative predictive value (NPP) of 94%, to predict early hypocalcemia, which was similar to the diagnostic accuracy of identification of the parathyroid glands using indocyanine green angiography (ICG) [15]. In a cohort of 63 patients, an ioPTH cutoff value of 70% decrease after thyroid gland removal predicted postoperative hypocalcemia with a sensitivity of 100%, specificity 82.9%, PPV 60.0% and NPV 100% [10]. However, the optimal cutoff values of ioPTH for predicting post-thyroidectomy hypoparathyroidism remain to be established.

We assessed the most reliable cutoff predictors of early and permanent hypoparathyroidism in a large series of patients undergoing total thyroidectomy.

## Materials and methods

### Design and patients

This was a retrospective analysis of a prospectively maintained database of patients undergoing surgery of the thyroid gland at the Unit of Endocrine Surgery of Hospital Universitari de Bellvitge in L’Hospitalet de Llobregat, Barcelona (Spain). For the purpose of the study, patients in whom a total thyroidectomy with and without central neck dissection was performed between January 2005 to May 2021 were selected. In addition to the type of surgical procedure, it was required to have available an intraoperative measurement of PTH according to the study protocol. Exclusion criteria were as follows: previous thyroid or parathyroid surgery, indication of concomitant parathyroidectomy due to primary hyperparathyroidism, lack of measurement of calcium serum levels within 24 h after the surgical procedure, poorly differentiated anaplastic carcinoma, and well-differentiated thyroid tumors stage T4b (any size tumors that has grown extensively beyond the thyroid gland). Patients with preoperative hypo- or hyperparathyroidism were also excluded. The primary endpoint of the study was the comparison of different cutoff values of ioPTH to identify those with the highest diagnostic efficacy for predicting postoperative early and permanent hypoparathyroidism.

The study was approved by the Institutional Review Board of Hospital Universitari de Bellvitge, and a written informed consent of all patients had been already obtained at the time of hospital admission for thyroid surgery.

### Procedures

Preoperatively, patients with 25-OH vitamin D deficiency (< 20 ng/mL) were treated with vitamin D supplementation, but none of the patients received calcium supplementation. Thyroid surgery was performed by a senior endocrine surgery (P.M.Ll.) using a standard protocol of total thyroidectomy, with attention to prevent incidental parathyroidectomy or injury/devascularization of the glands. As described by Bliss et al. [16] exposure of the thyroid gland is followed by careful dissection of the superior pole, paying special attention on identifying the external branch of the superior laryngeal nerve. “Capsular dissection” is performed after medial retraction of the gland, recurrent laryngeal nerves as well as parathyroid glands in orthotopic localizations are identified and left in situ.

The blood samples were obtained from a peripheral vein. The ioPTH levels were measured at the time of induction of anesthesia and 10 min after completion of surgery either after total thyroidectomy or after central neck dissection when this procedure was performed, and at 24 h postoperatively, using a chemiluminescent immunometric assay (reference limit 1.6–6.9 pmol/L) [17]. The percentage of ioPTH decline was calculated as (preoperative − postresection ioPTH)/preoperative ioPTH × 100. A colorimetric assay was used for measurement of corrected serum calcium levels at 24 h postoperatively and results were expressed as mmol/L (reference values 2.20–2.54 mmol/L). Criteria for the administration of calcium (1500 mg every 8 h) included the presence of symptoms of hypocalcemia or serum calcium levels at 24 h postoperatively < 1.8 mmol/L (7.20 mg/dL) in asymptomatic patients [15]. Vitamin D supplementation was introduced on the second postoperative day when the patient failed to normalize calcium levels after oral calcium administration. Permanent hypoparathyroidism was defined functionally as the need of calcium and/or vitamin D supplementation for 12 months after thyroid surgery to maintain calcium levels within the normal limit and the patient free of symptoms of hypocalcemia. For each patient, the following data were recorded: age, sex, preoperative diagnosis, need of central neck dissection, and presence of transient and permanent hypocalcemia.

### Statistical analysis

Categorical variables are expressed as frequencies and percentages, and continuous variables ad mean and standard deviation (SD). The diagnostic efficacy of ioPTH decline for the identification of transient hypocalcemia and permanent hypoparathyroidism was evaluated by the receiver operating characteristic (ROC) curve, with the area under the curve and the 95% confidence interval (CI). Three cutoff values were determined corresponding to a sensitivity of 95% and a specificity of 95%, and the optimal cutoff as the value with the highest percentage of patients correctly classified. The Statistical Package for the Social Sciences (SPSS) version 26 for Windows was used for data analysis.

## Results

During the study period, a total of 742 patients, 184 men and 558 women, with a mean age of 52 years (range 45–65 years) underwent total thyroidectomy and measurement of ioPTH. Preoperative diagnoses included thyroid cancer in 356 (48.0%) patients, benign thyroid nodule or goiter in 351 (47.3%), and hyperthyroidism in 35 (4.7%). Central neck dissection was performed in 312 (42.0%) patients. Postoperative hypocalcemia was diagnosed in 383 (51.6%) patients, which was transient hypocalcemia in 296 and permanent in 87 (11.7%).

**Fig. 1:**
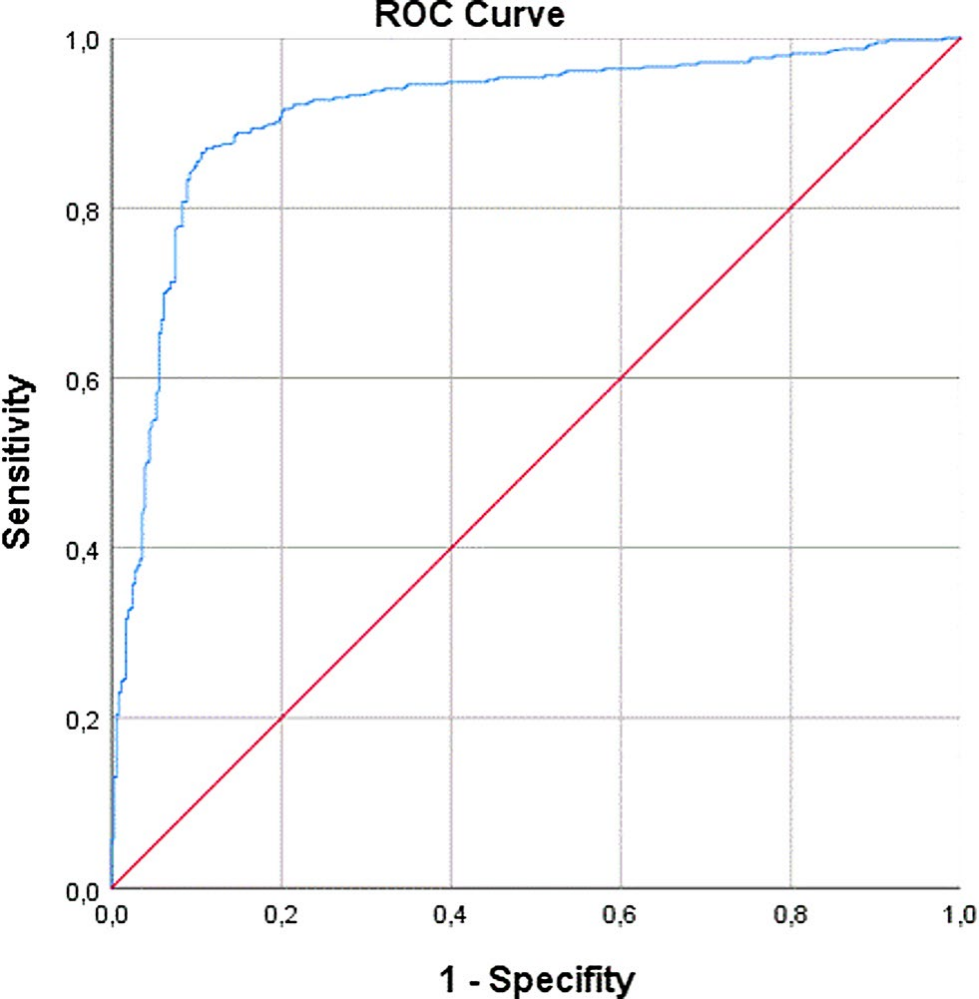
ROC curve for predicting transient hypocalcemia after total thyroidectomy based on the percentage of ioPTH decline with an optimal cutoff value of 62.5% (diagonal segments are produced by ties)

The mean decline in ioPTH levels was 54.6 ± 36%. In the analysis of postoperative hypocalcemia, the area under the ROC curve was 0.912 (95% confidence interval [CI] 0.890–0.935) (Fig. 1). The two cutoff values for a sensitivity of 95% and a specificity of 95% corresponded to a decline in ioPTH levels of 39.2% and 79.9%, respectively. The optimal cutoff value for the best overall diagnostic accuracy was decline of ioPTH of 62.5% (Table 1).

**Table 1:** Cutoff values of the percentage of ioPTH decline for predicting postoperative transient hypocalcemia after total thyroidectomy

ioPTH decline	Cutoff value	Diagnostic accuracy	
Sensitivity of 95%	39.2%	Specificity	55.7%
		Positive predictive value	69.6%
		Negative predictive value	91.3%
		Overall efficacy	76.0%
Specificity of 95%	79.9%	Sensitivity	55.1%
		Positive predictive value	92.1%
		Negative predictive value	66.5%
		Overall efficacy	74.4%
Optimal overall efficacy	62.5%	Sensitivity	87.5%
		Specificity	86.9%
		Positive predictive value	87.9%
		Negative predictive value	86.7%
		Overall efficacy	87.3%

**Fig. 2:**
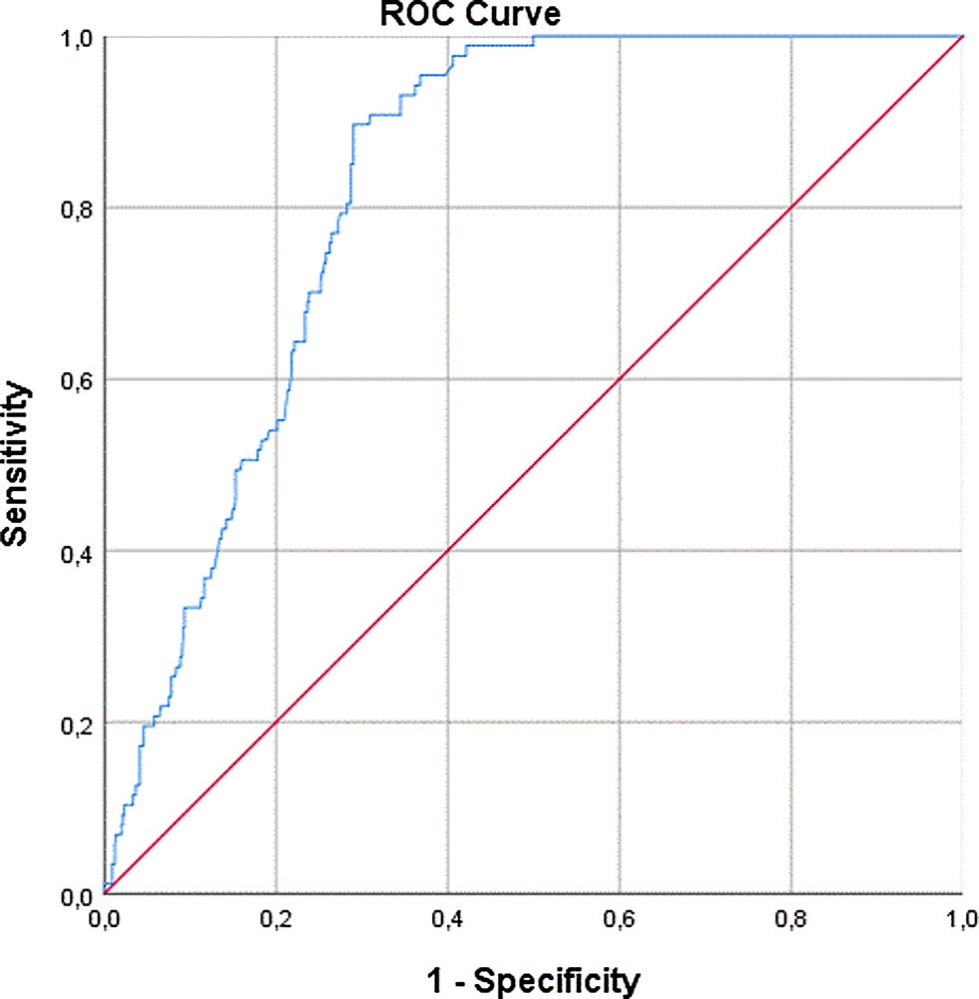
ROC curve for predicting permanent hypoparathyroidism after total thyroidectomy based on the percentage of ioPTH decline with an optimal cutoff value of 99% (diagonal segments are produced by ties)

In the analysis of permanent hypoparathyroidism, the area under the ROC curve was 0.824 (95% CI 0.792–0.857) (Fig. 2). The percentage of decline of ioPTH was 69% for a sensitivity of 95%, and 93.7% for a specificity of 95%. The optimal cutoff value for the best overall diagnostic accuracy was 99% (Tables 1 and 2).

**Table 2:** Cutoff values of the percentage of ioPTH decline for predicting postoperative permanent hypoparathyroidism after total thyroidectomy

ioPTH decline	Cutoff value	Diagnostic accuracy	
Sensitivity of 95%	69.0%	Specificity	63.2%
		Positive predictive value	25.6%
		Negative predictive value	99.0%
		Overall efficacy	67.0%
Specificity of 95%	93.7%	Sensitivity	80.5%
		Positive predictive value	34.7%
		Negative predictive value	89.9%
		Overall efficacy	86.3%
Optimal overall efficacy	99.0%	Sensitivity	1.1%
		Specificity	88.4%
		Positive predictive value	100%
		Negative predictive value	88.4%
		Overall efficacy	88.4%

## Discussion

In the present study, a simple formula based on results of ioPTH assay before starting thyroid surgery and after completion of total thyroidectomy/neck dissection was used to estimate the decline in ioPTH levels, which was used to identify cutoff points for 95% sensitivity, 95% specificity, and the optimal diagnostic efficacy for predicting postoperative hypoparathyroidism.

Based on the study findings, ioPTH was a good predictive factor for assessing the risk of early postoperative hypocalcemia, but its predictive capacity for permanent hypoparathyroidism, was less favorable. Permanent hypoparathyroidism after total thyroidectomy carries a large burden of disease [18], with a range of incidence between 1% and 14.5% in association with different factors, including the surgeon’s experience, central neck dissection, and heterogeneity among studies in the definition of clinical, biochemical and treatment criteria [19–23]. We used a functional clinical definition of permanent hypoparathyroidism as the need of calcium and/or vitamin D supplementation for 12 months after total thyroidectomy to maintain calcium levels within the normal limit and the patient free of symptoms of hypocalcemia. In this respect, the optimal cutoff value of an ioPTH decline of 99% was extremely displaced and had a lower clinical usefulness with a sensitivity of 1% only.

However, in the identification of transient hypocalcemia, the optimal cutoff value of 62.5% was reasonably adequate with an overall efficacy of 87% and balanced sensitivity, specificity and predictive values, with correct risk prediction in almost 9 out of 10 cases. In patients with an ioPTH decline ≤ 39%, there is a remote possibility of transient hypocalcemia and in carefully selected cases, total thyroidectomy may be safely undertaken as an ambulatory procedure [24]. Same-day thyroidectomy is a lower cost option for selected patients, particularly in specialty centers with experience in thyroid surgery. In a recent study of 9,571 same-day patients undergoing total thyroidectomy who were matched to 9,571 overnight patients, same-day patients had higher odds of treatment at a certified center, accredited teaching hospital, and high-volume hospital, with a median cost savings of $974 [25].

On the contrary, in the presence of an ioPTH decline ≥ 79.9%, there is a high risk of transient hypocalcemia, according to which early postoperative calcium supplementation would be justified. Development of permanent hypoparathyroidism is very unlikely in the presence of an ioPTH decline ≤ 63.2%, but patients with declines > 93.7% should be followed very closely since they may be considered high-risk patients for developing permanent hypoparathyroidism. Predictive values, however, should be interpreted with caution considering the occurrence of transient and permanent hypoparathyroidism in the study sample [26].

It is worthy to mention that Gupta et al. [11] conducted a similar study in a single-center prospective cohort 90 patients who underwent total thyroidectomy for benign as well as malignant pathologies of thyroid gland, using the same approach for measuring ioPTH decline as in our study. These authors found that the cutoff of an ioPTH decline of 86.21% combined with an ioPTH level of 9.3 pg/mL had maximum sensitivity of 100% and specificity of 97.5% for predicting permanent hypoparathyroidism. In our study, this combination was not evaluated, but a cutoff value of ioPTH decline of 93.7% showed 80.5% sensitivity and 95% specificity for predicting permanent hypothyroidism after total thyroidectomy. Assessment of the diagnostic accuracy of combining ioPTH as a percentage of decline and the ioPTH level is an interesting approach for further studies focused on identifying predictors of postthyroidectomy hypoparathyroidism.

Limitations of the study include the single-center characteristics and the retrospective design. A subgroup analysis of patients with and without neck dissection was not performed, although central neck dissection may be a factor potentially associated with a higher risk of postsurgical hypoparathyroidism and is an interesting aspect to be evaluated in further studies. Moreover, the effect of ioPTH decline as an independent factor associated with early and permanent hypoparathyroidism merits to be analyzed in a logistic regression model, but the present study was focused on the identification of optimal cutoff values of ioPTH decline and neither univariate or multivariate analyses were not performed.

## Conclusion

In large study population of 742 patients undergoing total thyroidectomy, the rates of transient and permanent hypoparathyroidism were 39.9% and 11.7%, respectively. The optimal cutoff value for predicting transient hypocalcemia was a decline of ioPTH level of 62.5%, but in the presence of a cutoff of ≥ 79.9%, the risk of early hypoparathyroidism is high and calcium supplementation would be indicated. Patients with declines > 93.7% should be followed very closely since they are high-risk for developing permanent hypoparathyroidism.

## Data Availability

No datasets were generated or analysed during the current study.

## References

[1] Kakava K, Tournis S, Papadakis G, Karelas I, Stampouloglou P, Kassi E et al (2016) Postsurgical hypoparathyroidism: a systematic review. Vivo 30(3):171–179 27107072

[2] Puzziello A, Rosato L, Innaro N, Orlando G, Avenia N, Perigli G et al (2014) Hypocalcemia following thyroid surgery: incidence and risk factors. A longitudinal multicenter study comprising 2,631 patients. Endocrine 47:537–542. 10.1007/s12020-014-0209-y 24563161 10.1007/s12020-014-0209-y

[3] Edafe O, Antakia R, Laskar N, Uttley L, Balasubramanian SP (2014) Systematic review and meta-analysis of predictors of post-thyroidectomy hypocalcaemia. Br J Surg 101(4):307–320. 10.1002/bjs.9384 24402815 10.1002/bjs.9384

[4] Eismontas V, Slepavicius A, Janusonis V, Zeromskas P, Beisa V, Strupas K et al (2018) Predictors of postoperative hypocalcemia occurring after a total thyroidectomy: results of prospective multicenter study. BMC Surg 18(1):55. 10.1186/s12893-018-0387-2 30092793 10.1186/s12893-018-0387-2 PMC6085643

[5] Díez JJ, Anda E, Sastre J, Pérez Corral B, Álvarez-Escolá C, Manjón L et al (2020) Permanent postoperative hypoparathyroidism: an analysis of prevalence and predictive factors for adequacy of control in a cohort of 260 patients. Gland Surg 9(5):1380–1388. 10.21037/gs-20-288 33224813 10.21037/gs-20-288 PMC7667118

[6] Xing T, Hu Y, Wang B, Zhu J (2019) Role of oral calcium supplementation alone or with vitamin D in preventing post-thyroidectomy hypocalcaemia: a meta-analysis. Med (Baltim) 98(8):e14455. 10.1097/MD.0000000000014455 10.1097/MD.0000000000014455 PMC6407934 30813146

[7] Bellantone R, Lombardi CP, Raffaelli M, Boscherini M, Alesina PF, De Crea C et al (2002) Is routine supplementation therapy (calcium and vitamin D) useful after total thyroidectomy? Surgery 132(6):1109–1113. 10.1067/msy.2002.128617 12490862 10.1067/msy.2002.128617

[8] Proye CA, Goropoulos A, Franz C, Carnaille B, Vix M, Quievreux JL et al (1991) Usefulness and limits of quick intraoperative measurements of intact (1–84) parathyroid hormone in the surgical management of hyperparathyroidism: sequential measurements in patients with multiglandular disease. Surgery 110(6):1035–1042 1745972

[9] Irvin GL 3rd, Dembrow VD, Prudhomme DL (1991) Operative monitoring of parathyroid gland hyperfunction. Am J Surg 162(4):299–302. 10.1016/0002-9610(91)90135-z 1683177 10.1016/0002-9610(91)90135-z

[10] Vaiman M, Mizrakli Y, Taha A, Gavriel H (2023) An individual approach to intraoperative parathyroid hormone measurement during total thyroidectomy. Am J Otolaryngol 45(2):104159. 10.1016/j.amjoto.2023.104159 38113776 10.1016/j.amjoto.2023.104159

[11] Gupta S, Chaudhary P, Durga CK, Naskar D (2015) Validation of intra-operative parathyroid hormone and its decline as early predictors of hypoparathyroidism after total thyroidectomy: a prospective cohort study. Int J Surg 18:150–153. 10.1016/j.ijsu.2015.04.074 25934417 10.1016/j.ijsu.2015.04.074

[12] Higgins KM, Mandell DL, Govindaraj S, Genden EM, Mechanick JI, Bergman DA et al (2004) The role of intraoperative rapid parathyroid hormone monitoring for predicting thyroidectomy-related hypocalcemia. Arch Otolaryngol Head Neck Surg 130(1):63–67. 10.1001/archotol.130.1.63 14732770 10.1001/archotol.130.1.63

[13] Lo CY, Luk JM, Tam SC (2002) Applicability of intraoperative parathyroid hormone assay during thyroidectomy. Ann Surg 236(5):564–569. 10.1097/00000658-200211000-00005 12409661 10.1097/00000658-200211000-00005 PMC1422613

[14] Lee DR, Hinson AM, Siegel ER, Steelman SC, Bodenner DL, Stack BC Jr (2015) Comparison of intraoperative versus postoperative parathyroid hormone levels to predict hypocalcemia earlier after total thyroidectomy. Otolaryngol Head Neck Surg 153(3):343–349. 10.1177/0194599815596341 26209077 10.1177/0194599815596341

[15] Moreno Llorente P, Francos Martínez JM, García Barrasa A (2020) Intraoperative parathyroid hormone measurement vs indocyanine green angiography of parathyroid glands in prediction of early postthyroidectomy hypocalcemia. JAMA Surg 155(1):84–85. 10.1001/jamasurg.2019.3652 31617879 10.1001/jamasurg.2019.3652 PMC6802248

[16] Bliss R, Gauger P, Delbridge L (2000) Surgeon’s Approach to the thyroid gland: Surgical anatomy and the importance of technique. World J Surg 24:891–897. 10.1007/s002680010173 10865032 10.1007/s002680010173

[17] Alía P, Moreno P, Rigo R, Francos JM, Navarro MA (2007) Postresection parathyroid hormone and parathyroid hormone decline accurately predict hypocalcemia after thyroidectomy. Am J Clin Pathol 127(4):592–597. 10.1309/J357LMD66E9X2505 17369135 10.1309/J357LMD66E9X2505

[18] Mitchell DM, Regan S, Cooley MR, Lauter KB, Vrla MC, Becker CB, Burnett-Bowie SA, Mannstadt M (2012) Long-term follow-up of patients with hypoparathyroidism. J Clin Endocrinol Metab 97(12):4507–4514. 10.1210/jc.2012-1808 23043192 10.1210/jc.2012-1808 PMC3513540

[19] Akgun IE, Unlu MT, Aygun N, Kostek M, Tufan AE, Yanar C et al (2022) The reality of hypoparathyroidism after thyroidectomy: which risk factors are effective? Single-center study. Sisli Etfal Hastan Tip Bul 56(2):262–269. 10.14744/SEMB.2022.24356 35990295 10.14744/SEMB.2022.24356 PMC9350047

[20] Ponce de León-Ballesteros G, Velázquez-Fernández D, Hernández-Calderón FJ, Bonilla-Ramírez C, Pérez-Soto RH, Pantoja JP, Sierra M et al (2019) Hypoparathyroidism after total thyroidectomy: importance of the intraoperative management of the parathyroid glands. World J Surg 43(7):1728–1735. 10.1007/s00268-019-04987-z 30919027 10.1007/s00268-019-04987-z

[21] Sitges-Serra A (2017) The PGRIS and parathyroid splinting concepts for the analysis and prognosis of protracted hypoparathyroidism. Gland Surg 6(Suppl 1):S86–S93. 10.21037/gs.2017.07.16 29322026 10.21037/gs.2017.07.16 PMC5756749

[22] Orloff LA, Wiseman SM, Bernet VJ, Fahey TJ 3rd, Shaha AR, Shindo ML et al (2028) American Thyroid Association Statement on Postoperative Hypoparathyroidism: diagnosis, prevention, and management in adults. Thyroid 28(7):830–841. 10.1089/thy.2017.0309 10.1089/thy.2017.0309 29848235

[23] Díez JJ, Anda E, Sastre J, Pérez Corral B, Álvarez-Escolá C, Manjón L et al (2019) Prevalence and risk factors for hypoparathyroidism following total thyroidectomy in Spain: a multicentric and nation-wide retrospective analysis. Endocrine 66(2):405–415. 10.1007/s12020-019-02014-8 31317524 10.1007/s12020-019-02014-8

[24] Compton RA, Simmonds JC, Dhingra JK (2020) Total thyroidectomy as an ambulatory procedure in community practice. OTO Open 4(3):2473974X20957324. 10.1177/2473974X20957324 33062910 10.1177/2473974X20957324 PMC7534086

[25] Finn CB, Sharpe JE, Krumeich LN, Ginzberg SP, Soegaard Ballester JM, Tong JK (2024) The use and costs of same-day surgery versus overnight admission for total thyroidectomy: a multi-state, all-payer analysis. Surgery 175(1):207–214. 10.1016/j.surg.2023.06.051 37989635 10.1016/j.surg.2023.06.051 PMC10870294

[26] Carter JV, Pan J, Rai SN, Galandiuk S (2016) ROC-ing along: evaluation and interpretation of receiver operating characteristic curves. Surgery 159(6):1638–1645. 10.1016/j.surg.2015.12.029 26962006 10.1016/j.surg.2015.12.029

